# Congenital Epulis: A Rare Benign Jaw Tumor Of Newborn

**Published:** 2012-04-01

**Authors:** Mahavir Singh, KN Rattan, Babita Rani

**Affiliations:** Department of Paediatric surgery, Pt BD Sharma Postgraduate Institute of Medical Sciences Rohtak, India; 1 Department of Community medicine, Pt BD Sharma Postgraduate Institute of Medical Sciences Rohtak, India

**Dear Sir,**

A congenital epulis is a rare benign jaw tumor of neonate and is also known as “congenital gingival granular cell tumor”. The Greek word ‘‘epulis’’ means ‘‘swelling of the gingiva’’. It was first described in 1871 by Neumann; hence the alternative name is Neumann’s Tumor [1]. It usually presents at birth with an obvious mass arising from the gingival mucosa of the maxilla or mandible. It commonly presents in neonates, although prenatal diagnosis with ultrasound has been reported as early as 26 weeks gestation [2]. The lesion usually arises from the gingival mucosa of the maxilla or mandible (maxillary/mandibular ratio 3:1). However, it has also been described on the tongue [3].

There is a marked female preponderance of 10:1. This lesion is often pedunculated, flesh-pink colored, firm with a smooth or lobulated surface and in general solitary. However, multiple lesions may also occur in up to 10% [1]. The size of the mass varies from a few millimeters to few cm in diameter. Large lesions may interfere with respiration, feeding or adequate closure of the mouth. The lesions commonly interfere with feeding. Its etiology, histopathogenesis and natural history is still unclear [4]. The diagnosis is usually made on clinical grounds alone, although difficulties may arise when the size of the lesion is small, or the index of suspicion is low. Although few cases of spontaneous regression have been reported, the recommended treatment is surgical excision under local or general anaesthesia [5]. There are no reports of recurrence, or malignant change.

A 2.5-kg neonate born by normal term vaginal delivery at home was brought for something protruding from the mouth causing feeding problem. On examination, a round, 3x2 cm pedunculated, smooth, pink-colored soft tissue mass covered by mucous membrane was protruding from the mouth, attached to the maxillary incisor region (Fig. 1). Mass was firm in consistency and was not tender on palpation. Adjacent tissue appears normal on examination. The lesion prevented the patient from feeding but did not interfere with respiration. A differential diagnosis of congenital epulis, hemangioma, and teratoma was made. She was booked for excision of this mass under general anesthesia. Mass was removed with an elliptical incision to the peduncles and hemostasis was achieved with diathermy. Postoperative recovery was uneventful. The child was breastfeeding the day after surgery, and discharged home the following day. Excised tissue was sent for histopathological examination. Its microscopic examination showed a benign tumor composed of sheets of closely packed, large, polygonal cells, with abundant eosinophilic granular cytoplasm with small central nuclei consistent with histopathology of congenital epulis.

**Figure:**
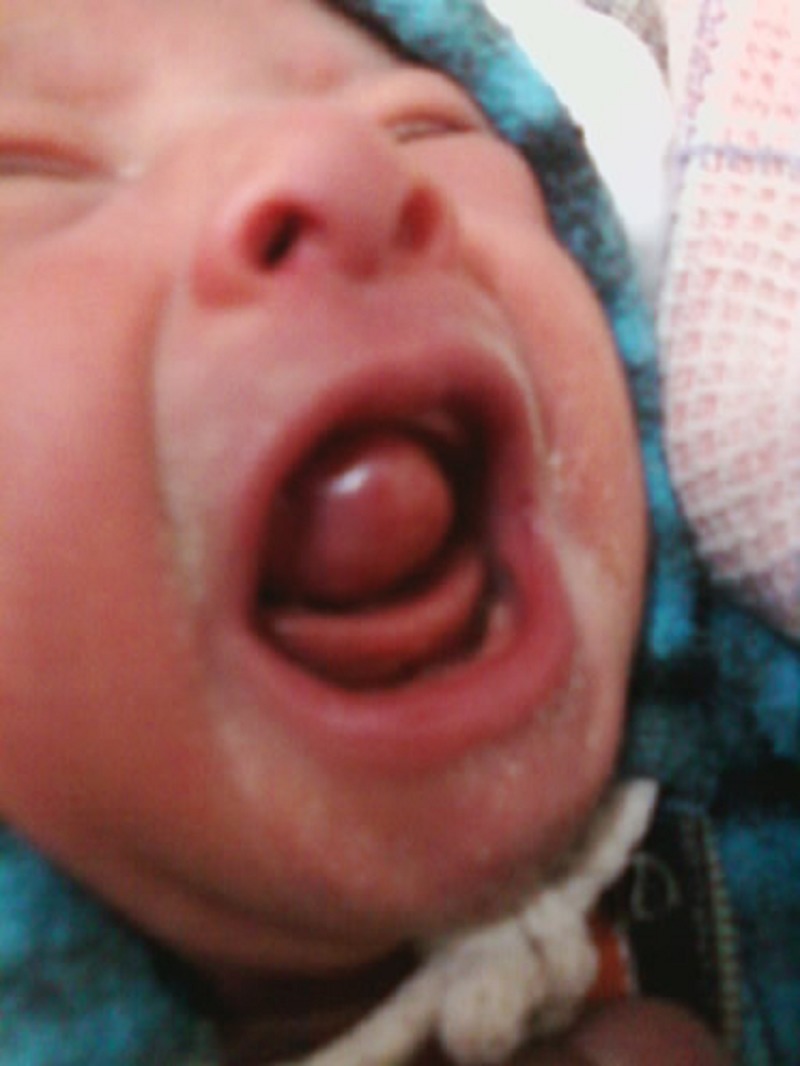
Figure 1: Epulis.

Histologically, congenital epulis shows remarkable similarity with the more common Granular Cell Tumors (GCTs). The clinical presentation of this congenital tumor can be distressing due to its size and aggressive appearance, it is important for the attending pediatricians, pediatric surgeon to be aware of this rare congenital tumor.

## Footnotes

**Source of Support:** Nil

**Conflict of Interest:** None declared

